# Worldwide research trends in Crohn’s disease treatment over the past 2 decades: a bibliometric analysis

**DOI:** 10.3389/fphar.2024.1441785

**Published:** 2024-10-08

**Authors:** Likang Xu, Jinzhao Zou, Chao Sun, Gong Chen, Sujun Gao

**Affiliations:** ^1^ Department of Clinical Medicine, Medical College, Yangzhou University, Yangzhou, China; ^2^ Department of Medical Imaging, Northern Jiangsu People’s Hospital, Clinical Medical College of Yangzhou University, Yangzhou, China; ^3^ Digestive Department of Northern Jiangsu People’s Hospital, Clinical Medical College of Yangzhou University, Yangzhou, Jiangsu, China

**Keywords:** Crohn’s disease, bibliometric analysis, web of science, treatment, citespace

## Abstract

**Background:**

The treatment of Crohn’s disease (CD) has received widespread attention in clinical practice, but there is currently a lack of quantitative evaluation of the literature published in this field. This study aimed to describe the development trends and research hotspots of CD treatment through bibliometric analysis.

**Methods:**

Publications related to CD treatment published from 2004 to 2023 were searched in the WoSCC. Microsoft Office Excel 2021 was used for the analysis and visualization of the annual number of publications. CiteSpace was used to visualize the collaboration networks of authors, institutions, and countries, as well as to construct a reference timeline visualization map and identify keywords with the strongest citation bursts.

**Results:**

The bibliometric analysis included 25,608 publications between 2004 and 2023. The most productive year was 2021. The United States of America (n = 7,891) and the University of California System (n = 939) are the country and institution with the most published papers, respectively. Among the 97,564 authors, Peyrin-Biroulet, Laurent (n = 424) published the most articles. The core journals were Inflammatory Bowel Diseases, Journal of Crohns and Colitis, Alimentary Pharmacology and Therapeutics, etc. The timeline view showed that “#5 JAK Inhibitor” was the most recent topic. The keywords that burst and persist from 2020 to 2023 include “ustekinumab” and “vedolizumab”.

**Conclusion:**

An increasing number of researchers are dedicating their efforts to exploring the treatment of CD, with the United States making the largest contribution to this field. Currently, the research hotspots predominantly involve drug therapy including ustekinumab, vedolizumab, and JAK inhibitors. Our study provides valuable information for scholars studying CD treatment.

## 1 Introduction

Crohn’s disease (CD) is a chronic inflammatory bowel disease (IBD) that is commonly characterized by diarrhea and abdominal pain, along with fatigue, fever, weight loss, and anemia (Roda et al., 2020). It is characterized by lifelong recurrence and progressive destruction. As the disease progresses, the gastrointestinal damage caused by CD gradually worsens, which can lead to serious complications such as intestinal obstruction, abscess and perforation (Baumgart and Sandborn, 2012). These conditions not only severely affect patients’ psychological health, quality of life, and cause significant personal and economic losses but also increase the risk of cancer (Cantoro et al., 2023).

The foundational principle of CD treatment involves employing diverse strategies to mitigate inflammation of the intestinal mucosa, manage clinical symptoms, and achieve mucosal healing (Torres et al., 2017). Drug management, surgical interventions, and health education constitute the most critical dimensions of CD treatment. It is mainly managed with immunomodulators and biologic agents. Immunomodulators, including thiopurines and methotrexate, are used as monotherapies for the maintenance or remission of CD and to prevent the development of antidrug antibodies (De Boer et al., 2018; Crepaldi et al., 2023). Biologic agents are among the most effective therapies for inducing and maintaining remission in patients with CD (D'Haens and Van Deventer, 2021). Surgical indications can be divided into abdominal CD and perianal CD, with emergent and non-emergent considerations, respectively (Dolinger et al., 2024). Emergent indications for abdominal Crohn’s disease include acute bowel obstruction and intestinal perforation, while non-emergent indications are medically refractory disease. For perianal Crohn’s disease, emergent indications include pelvic sepsis, and non-emergent indications include complex fistulae and perianal abscesses. Research also indicates that reducing smoking habits contributes to a decreased necessity for surgical interventions (Dittrich et al., 2020).

There have been several recent reviews on CD treatment (Neurath, 2024; Wu Q. et al., 2024; Zhang et al., 2024). They focus on depicting the latest research advancements and the present scenario, and do not analyze countries, institutions, authors or journals, let alone hotspot changes. Bibliometric analyses offer a quantitative analysis of authors, countries, institutions, keywords, and references in publications within a field (Chen et al., 2024). It has evolved into a vital instrument for tracking research trends and forecasting future directions and has been extensively employed across medical and other fields (He et al., 2023; Zhou et al., 2023). Alpaslan Karabulut and Muhammed Kaya conducted a bibliometric analysis on the research trends and global scholarly contributions to CD from 1980 to 2022, suggesting that CD treatment will be a primary research emphasis in the field (Karabulut and Kaya, 2023). Some studies also use bibliometrics to analyze nutrition and biologic agents, finding that research mainly focuses on exclusive enteral nutrition and anti-TNF drugs (Shen et al., 2022; Shakhshir and Zyoud, 2023). To the best of our knowledge, no bibliometric analysis has focused specifically on CD treatment.

The purpose of this study was to utilize bibliometric methods to evaluate the developmental framework, present conditions and future directions of the CD treatment field, thereby assisting researchers in understanding the primary research trends and potential opportunities within the field.

## 2 Materials and methods

### 2.1 Data sources and retrieval strategies

The data were extracted on 22 January 2024. To systematically review the research literature on CD treatment over the past 20 years, the Web of Science core collection (WoSCC) database was used (Peng et al., 2024). The search strategy was as follows: TS= (“Crohn Disease” OR “Crohns Disease” OR “Crohn’s Disease”) AND TS= (“therap*” OR “treatment”) AND FPY= (2004–2023).

**FIGURE 1:**
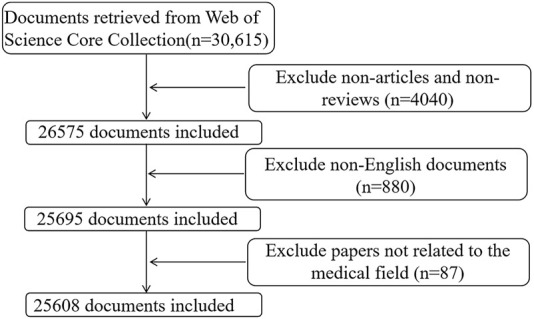
Flow chart of data collection in this study.

The preliminary search yielded a total of 30,615 documents. After excluding non-articles/non-reviews (n = 4,040) and non-English documents (n = 880), 25,695 documents met the preliminary standards. Eighty-seven papers were excluded manually because they were not related to the medical field, including zoology and veterinary science. A total of 25,608 documents were included in the bibliometric analysis. The data for this study were exported in “plain text” format from the “Full Record and Cited References” section of the WoS platform. Figure 1 depicts the retrieval and screening process in detail. Additionally, citation reports for the top 10 most productive countries, institutions, and authors and the top 15 most productive journals from the WoSCC were recorded.

### 2.2 Data processing and analysis

The “remove duplicates” function of CiteSpace software, confirmed the absence of duplicate documents. Keyword merging primarily included synonyms, aliases, and singular/plural forms to maintain the integrity of the research (Wang et al., 2024). For example, “therapies”, “treatment”, and “therapy” are unified as “therapy”.

Microsoft Office Excel 2021 was used to visualize the annual number of publications and the cumulative number of publications. CiteSpace (6.2.R6 advanced) was used to visualize the analysis of cooccurrences among institutions and countries, to construct a reference timeline visualization map, and to identify keywords with the strongest citation bursts.

The highly cited index (H-index) is a hybrid quantitative index based on the WOS that can assess the quantity and level of a researcher’s academic achievements (Chen et al., 2024). The average number of citations per publication (AC/P) refers to the average number of citations a scholar, research institution, or country receives over a certain period, which is used to measure the attention their research receives (Wang et al., 2024). In the reference timeline visualization map, the modularity value (Q value) and the mean silhouette value (S value) are used to evaluate the results of clustering and the rationality of clustering. A Q value greater than 0.3 indicates a significant cluster structure, while an S value greater than 0.7 signifies that the clustering result is reliable and satisfactory (Wang et al., 2023; Ye et al., 2023).

### 2.3 Charts interpretation

In the collaboration network generated by CiteSpace, each node represents a specific parameter. The size of a node reflects its frequency of occurrence. The color of a node indicates its average time of first appearance or related time. The lines between nodes show the degree of collaboration or cross-association among them. A notable advantage of network visualization is its ability to quickly reveal the frequency distribution of nodes and accurately highlight key and influential nodes (Wu Z. et al., 2024).

## 3 Results

### 3.1 Annual publications

**FIGURE 2:**
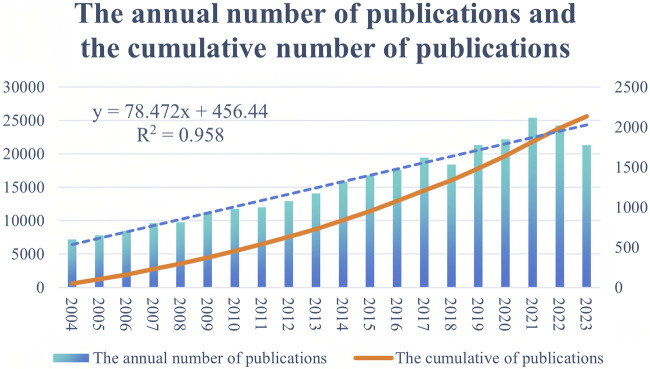
Annual trends of global publication outputs in Crohn’s disease treatment studies from 2004 to 2023.

A total of 25,608 research documents related to CD treatment were analyzed, including 18,446 articles and 6,642 reviews. The annual and cumulative numbers of publications are depicted in Figure 2, which shows a consistent increase in the field of CD treatment from 2004 to 2021, peaking in 2021. A linear regression analysis (*R* ^2^ = 0.958) demonstrated a continuous linear growth trend in the CD treatment group.

### 3.2 Analysis of the countries and institutions

**FIGURE 3:**
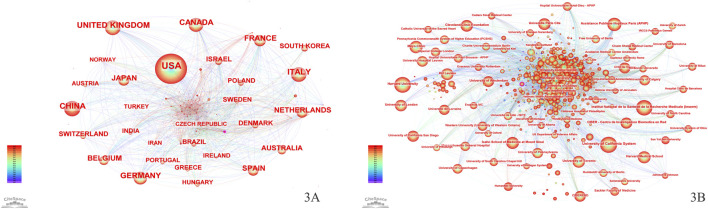
**(A)** Country/region network visualization. **(B)** Institution network visualization.

More than 100 countries have contributed to global efforts to advance CD treatment (Figure 3A). Table 1 lists the top 10 countries by the number of publications. The United States of America (United States) has the greatest number of publications (7,891), accounting for 30.81%, followed by the United Kingdom (2,549) and Italy (2,515). For the H-index, the United States (261), the United Kingdom (173), and Germany (165) are the top three countries.

**TABLE 1:** The top 10 most productive countries in Crohn’s disease treatment research.

Rank	Country	Count	Percentage (%)	H-index	AC/P
1	United States	7,891	30.81	261	55.95
2	United Kingdom	2,549	9.95	173	66.84
3	Italy	2,515	9.82	132	39.79
4	China	2,435	9.51	96	23.17
5	Canada	2,069	8.08	163	66.04
6	Germany	1,929	7.53	165	67.23
7	France	1,661	6.49	157	73.49
8	Japan	1,359	5.31	92	30.68
9	Spain	1,311	5.12	111	46.05
10	Netherlands	1,280	5.00	129	63.24

Over 70,000 institutions worldwide have participated in research on the treatment of CD (Figure 3B). Table 2 presents the institutions with the top 10 publications, where the University of California System ranks first (939 publications), followed by Harvard University (912 publications) and the Institut National de la Sante et de la Recherche Medicale (Inserm) (832 publications). Among the top 10 institutions by the number of publications, half are from the United States, with three located in France. Regarding the H-index, the University of California System leads with a score of 129, followed by KU Leuven (126) and Harvard University (118).

**TABLE 2:** The top 10 most productive institutions in Crohn’s disease treatment research.

Rank	Institutions	Count	Country	H-index	AC/P
1	University of California System	939	United States	129	80.62
2	Harvard University	912	United States	118	78.64
3	Institut National de la Sante et de la Recherche Medicale (Inserm)	832	France	113	74.72
4	Assistance Publique Hopitaux Paris (APHP)	621	France	105	81.78
5	Mayo Clinic	614	United States	103	90.32
6	Icahn School of Medicine at Mount Sinai	611	United States	102	90.57
7	KU Leuven	604	Belgium	126	120.29
8	Universite Paris Cite	597	France	100	86.49
9	Harvard Medical School	590	United States	103	80.34
10	University of London	582	United Kingdom	93	67.44

### 3.3 Analysis of authors

Among the 97,564 authors, the top 10 most productive authors published 2,621 articles, accounting for 10.2% (Table 3). Peyrin-Biroulet, Laurent contributed the most articles (424, accounting for 1.66%), followed by Sandborn, William, and Vermeire, Severine. Sandborn, William boasts the highest H-index (112) among all authors. It is worth mentioning that Rutgeerts, Paul represents the highest quality of his research, with an average number of citations per article of 218.24.

**TABLE 3:** The top 10 most productive authors in Crohn’s disease treatment research.

Rank	Authors	Count	H-index	Citations	AC/P
1	Peyrin-Biroulet, Laurent	424	82	28962	68.31
2	Sandborn, William	384	112	58426	152.15
3	Vermeire, Severine	324	93	31732	97.94
4	Colombel, Jean Frederic	303	97	41947	138.44
5	Danese, Silvio	265	70	19943	75.26
6	Rutgeerts, Paul	215	96	46921	218.24
7	Van Assche, Gert	181	76	25066	138.49
8	Gisbert, Javier P	180	51	8700	48.33
9	Loftus, Edward V	175	61	16163	92.36
10	Panaccione, Remo	170	63	19642	115.54

### 3.4 Analysis of journals

There are 2,636 journals that have published articles related to the treatment of CD. The 15 most productive journals are listed in Table 4. Inflammatory Bowel Diseases had the highest number of articles (1,932), followed by the Journal of Crohn’s and Colitis (1,012) and Alimentary Pharmacology and Therapeutics (773). The IF and JCR quartiles were obtained from Journal Citation Reports (JCR). Inflammatory Bowel Diseases has published the greatest number of articles on CD treatment, indicating that it is currently the most favored journal in the domain of CD treatment. Gastroenterology had the greatest impact (IF = 29.4006). Researchers may consider these journals to be key references and reliable sources of information.

**TABLE 4:** The 15 most productive journals in CD treatment research.

Rank	Journal	Count	IF2022	JCR quartile
1	Inflammatory Bowel Diseases	1,932	4.9000	Q2
2	Journal of Crohns and Colitis	1,012	8.0000	Q1
3	Alimentary Pharmacology and Therapeutics	773	7.6001	Q1
4	World Journal of Gastroenterology	711	4.2997	Q2
5	Digestive Diseases and Sciences	508	3.1000	Q3
6	Clinical Gastroenterology and Hepatology	408	12.6005	Q1
7	Journal of Pediatric Gastroenterology and Nutrition	386	2.8997	Q3
8	American Journal of Gastroenterology	358	9.8003	Q1
9	Scandinavian Journal of Gastroenterology	353	1.9002	Q4
10	Gastroenterology	339	29.4006	Q1
11	Gut	305	24.500	Q1
12	European Journal of Gastroenterology and Hepatology	293	2.100	Q4
13	Plos One	289	3.700	Q2
14	Digestive and Liver Disease	285	4.500	Q2
15	Digestive Diseases	242	2.3000	Q4

### 3.5 Analysis of keywords

**FIGURE 4:**
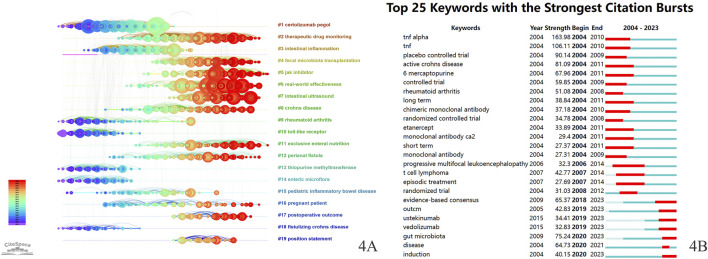
**(A)** Reference timeline visualization map for Crohn’s disease treatment (Q = 0.81, S = 0.9215). **(B)** Top 25 keywords with the strongest citation bursts. The light green lines indicate that a keyword has not yet appeared. The dark green lines represent the time period during which a keyword was researched. The red lines represent the time span of keyword bursts.

The reference timeline visualization map was constructed using CiteSpace (Figure 4A), with references clustered based on “Title words”. The clusters are numbered from 1 to 19, with a smaller number encompassing a greater number of references. In the reference timeline visualization map, the horizontal axis represents the passage of time, with each horizontal line displaying a label for a cluster. The clusters were: “#1 certolizumab pegol”, “#2 therapeutic drug monitoring”, “#3 intestinal inflammation”, “#4 fecal microbiota transplantation”, “#5 JAK inhibitor”, “#6 real-world effectiveness”, “#7 intestinal ultrasound”, “#8 Crohn’s disease”, “#9 rheumatoid arthritis”, “#10 toll-like receptor”, “#11 exclusive enteral nutrition”, “#12 perianal fistula”, “#13 thiopurine methyltransferase”, “#14 enteric microflora”, “#15 pediatric inflammatory bowel disease”, “#16 pregnant patient”, “#17 postoperative outcome”, “#18 fistulizing Crohn’s disease”, and “#19 position statement”.

Burst detection serves to identify keywords with a rapid increase in frequency over a short duration, thus uncovering research hotspots and cutting-edge areas. Figure 4B illustrates the bursts of the top 25 keywords, with the red sections of the blue lines representing the burst durations of the keywords. The strongest burst intensity was observed for TNF-α, followed by TNF and the placebo. Additionally, keyword bursts continuing into 2023 included ustekinumab, vedolizumab, gut microbiota, induction, and evidence-based consensus.

Based on Figures 4A, B, we can categorize CD treatment research into approximately three periods. The first period (2004–2011) included key keywords such as “mercaptopurine”, “monoclonal antibody ca2”, “infliximab”, “certolizumab pegol”, “etanercept”, “pediatric inflammatory bowel disease”, and “pregnant patient”. The second period (2012–2018) included the main keywords “therapeutic drug monitoring (TDM)”, “exclusive enteral nutrition (EEN)”, “episodic treatment”, and “fecal microbiota transplantation (FMT)”. The third period (2019–2023) featured the principal keywords “ustekinumab”, “vedolizumab”, and “JAK Inhibitor”.

## 4 Discussion

In this study, we analyzed 25,608 documents related to CD treatment over 20 years from the WoSCC. The annual number of publications in this field has rapidly increased, exceeding 2,000 articles in 2021 and reaching a peak. Peyrin-Biroulet, Laurent has the highest number of publications. Sandborn, William has the highest H-index (112) in the field. Vermeire, Severine, Rutgeerts, Paul, and Van Assche, Gert all come from KU Leuven in Belgium and they maintain a close collaborative relationship in the field of CD. There could be two possible reasons for this phenomenon: ① The team has conducted extensive studies on the treatment of CD, leading to a high number of publications; ② The authors mentioned above have collaborated and co-authored in numerous articles. Additionally, the countries of the authors do not necessarily reflect the geographical distribution of the patient populations involved in the studies. To address this disparity, it is crucial to encourage researchers to collaborate with regions that have a high prevalence of CD. Such collaborations can ensure that studies are more representative of the global patient population, enhance the applicability of the research findings across diverse settings, and ultimately contribute to clinical practices worldwide. The United States of America is the most productive country. It contributes 30.81% of the total publications with an H-index of 261 and demonstrates its leading position and influence in the field of CD treatment. France with a greatest AC/P (78.49) demonstrates the effective backing of its scientific policies and environment, alongside the significant influence of its research outputs. An important factor contributing to France’s high academic influence in the field of CD treatment is the close collaboration among the APHP clinical hospitals in the Paris, the national research network INSERM, and various universities. This multi-party cooperation among hospitals, research institutions, and universities has jointly promoted advancements in CD treatment research, which reflects excellent collaborative relationships and resulting in fruitful academic achievements. We have found that countries leading in CD research are predominantly economically developed nations. The economic prosperity of these countries provides both a solid foundation and ample funding for CD research, advancing them to the forefront of scientific and technological innovation. Another important consideration is that many registration studies are conducted to fulfill the regulatory requirements of agencies such as FDA and EMA. Therefore, it is not unexpected to observe a majority of these studies being performed in the United States and European countries. For more recent pharmaceutical products, global registration studies have increasingly included countries outside the United States and EMA jurisdictions, provided that local regulatory authorities agree with the FDA and EMA agreed protocols. As the incidence of CD increases, particularly in Western countries, there is a rising demand for effective treatments. This increasing disease burden not only heightens the need for therapeutic research but also strengthens international collaborations in CD treatment. Such international cooperation enables the sharing of research outcomes, standardization of treatment protocols, and collective addressing of the challenges posed by CD. This shift in global research priorities could profoundly influence future research trajectories and clinical practices, promoting the worldwide standardization of treatment methods for CD. However, despite their high output, Asian countries such as China and Japan exhibit lower H-index and AC/P compared to their Western countries, suggesting a disparity in high-impact, high-quality research. Enhanced international cooperation, extending beyond the United States and European countries, is essential to elevate the global impact of research findings and improve clinical practices worldwide (Shen et al., 2022). Timeline view and keyword burst analyses adeptly depict current research focal points and nascent trends over designated time spans (Wang et al., 2023). Research on CD treatment can be divided into three periods. In the first period (before 2011), research efforts were primarily focused on analyzing the application and safety of anti-TNF drugs in CD. The keywords included mercaptopurine, monoclonal antibody ca2, infliximab, certolizumab pegol, etanercept, pediatric inflammatory bowel disease, and pregnant patient. Monoclonal antibody ca2, infliximab, certolizumab pegol, and etanercept are anti-TNF drugs. The monoclonal antibody ca2 was later named infliximab and approved by the FDA in 1998 for refractory CD (Kornbluth, 1998). The first observational study of anti-TNF drugs involved the treatment of 10 patients with refractory CD using the monoclonal antibody ca2, which reduced inflammation by blocking TNF, showing remarkably significant clinical efficacy (Van Dullemen et al., 1995). Infliximab has been continuously employed to date due to its effectiveness in treating patients with moderate-to-severe CD. Certolizumab pegol is a pegylated antibody fragment that can bind to TNF(Lodhia and Rao, 2022). It may significantly increase the success rate of maintaining clinical remission and response within 26 weeks (Okabayashi et al., 2022). However, the use of certolizumab pegol is limited to fewer countries. Etanercept is an anti-TNF medication approved for the management of rheumatoid arthritis. Studies have explored the application of etanercept for treating CD, but it is ineffective in treating CD and may even increase the risk of CD (Korzenik et al., 2019; D'Haens and Van Deventer, 2021). Additionally, adalimumab, a common anti-TNF drug, is recommended as a monotherapy for the induction and maintenance of remission in moderate-to-severe CD. For patients without prior experience with biologic therapies, it is advised to use adalimumab alone rather than in combination with immunomodulators (Gordon et al., 2024). Mercaptopurine is an effective immunomodulator and is frequently used in the maintenance remission of CD or in combination therapy with anti-TNF drugs to reduce the development of anti-drug antibodies, yet its ability to induce clinical remission is slow, rendering it unsuitable for rapid disease management (De Boer et al., 2018). Moreover, mercaptopurine can induce systemic discomfort and gastrointestinal side effects, which leads to its early discontinuation. Approximately 60% of IBD patients halt their thiopurine maintenance therapy, predominantly ceasing thiopurine medication within the initial months of treatment (Jharap et al., 2010). In the later part of the first period, the emergence of new drugs inevitably necessitated identifying appropriate patient groups, leading scholars to conduct more research on the safety and efficacy of drugs for different populations, especially for patients with pediatric inflammatory bowel disease and pregnant patients (Kammermeier et al., 2023; Wieringa et al., 2023).

In the second period (2012–2018), the hotspot of CD treatment was the optimization of CD treatment plans. The keywords included TDM, EEN, episodic treatment, and FMT. TDM monitors drug concentrations and anti-drug antibodies in serum to guide dosage adjustments, aiming to improve efficacy and reduce the risk of complications, which has become the key in optimizing anti-TNF therapy for CD (Martins et al., 2022). Episodic treatment is administered during CD flare-ups or worsening conditions and is stopped when CD is in remission. Compared to episodic treatment with infliximab, maintenance therapy results in greater improvement of mucosal ulcers and higher rates of mucosal healing, achieving higher sustained remission rates and lower immunogenicity (Rutgeerts et al., 2006; Jauregui-Amezaga et al., 2011). Patients with EEN typically receive a comprehensive nutritional liquid diet for a duration of 6–8 weeks excluding other foods. EEN is considered safe, cost-efficient, and effective and has been established as the primary treatment for mild to moderate active CD in children, demonstrating its utility in adult CD therapy (Ruemmele et al., 2014; Ge et al., 2019; Adolph and Zhang, 2022; Hashash et al., 2024). EEN might operate through impacting the intricate interaction mechanisms between host mucosal immune responses and the luminal environment (Melton et al., 2023). ENN can also induce microbiota alterations by gut bacteria-mediated histidine biosynthesis to promote the remission of CD (Zeng et al., 2024). FMT is defined as the infusion of feces from healthy donors into the gastrointestinal tract of recipients to treat disease-associated gut dysbiosis (Lopetuso et al., 2023). FMT significantly enhances microbial diversity in patients’ intestines, restoring healthy gut microbiota for CD treatment (Vaughn et al., 2016).

In the third period (2019–2023), studies targeted alternative pathways to anti-TNF agents. The keywords included ustekinumab, vedolizumab, and JAK inhibitors. Ustekinumab is a monoclonal antibody targeting the P40 subunit of IL-12 and IL-23 that prevents IL-12 and IL-23 from participating in the inflammatory process and is used to induce and maintain treatment of refractory CD (Sandborn et al., 2012; Feagan et al., 2016). Currently, ustekinumab has demonstrated good efficacy and safety in both short-term and long-term use in treating CD (Kubesch et al., 2019). In patients with moderate-to-severe CD, ustekinumab and adalimumab show similar efficacy as induction and maintenance therapies, with no significant differences in safety or remission rates (Gordon et al., 2024).Vedolizumab specifically binds to α4β7 integrin and prevents immune cells from migrating to the intestinal mucosa through integrin, which reduces CD inflammation (Luzentales-Simpson et al., 2021). Vedolizumab is also recommended for the induction and maintenance treatment of moderate-to-severe CD. It effectively induces clinical remission and maintains steroid-free clinical remission, with a high safety profile (Honap et al., 2023; Gordon et al., 2024). This specificity allows for better disease management through reduced systemic complications, impacting long-term patient safety and quality of life. The administration of biologic agents, including ustekinumab and vedolizumab, imposes a burden on the healthcare system and the inconveniences of patients seeking treatment, including intravenous or subcutaneous injection, biologic agent storage and monitoring complexities. Furthermore, some patients may not respond to these treatments or may lose responsiveness over time, and treatment may be discontinued due to adverse reactions (Nielsen et al., 2023). As small-molecule medications, JAK inhibitors interfere with the STAT signaling pathway and have broad and potent anti-inflammatory effects. Thus, the administration of biologic agents is limited. The JAK protein family has four members: JAK1, JAK2, JAK3, and Tyrosine kinase 2 (Tyk2), which are intracellular tyrosine kinases (Yamaoka et al., 2004). JAK inhibitors bind to the kinase domain of JAK proteins and inhibit their activation, thereby preventing downstream STAT phosphorylation and translocation to the nucleus, thus interrupting the activation of multiple cytokine pathways to regulate immunity and inflammation (Dell’Avalle et al., 2022). JAK inhibitors have a fast onset of action, rapid metabolism, and lack immunogenicity, which may make them more suitable for biological treatment cycles in CD. For the majority of patients previously treated with at least one biologic agent, the JAK1 inhibitor upadacitinib has proven effective in managing moderate to severe CD (Abreu, 2023). Upadacitinib, the only recommended JAK inhibitor, is strongly recommended for inducing and maintaining remission in patients with moderate-to-severe CD (Abreu, 2023). Filgotinib preferentially binds to JAK1. In a phase II clinical trial, it was shown to induce clinical remission in active CD patients with acceptable safety (Vermeire et al., 2017). Tofacitinib, an inhibitor of JAK1 and JAK3, has superior effects on the induction and maintenance treatment of ulcerative colitis. Intriguingly, tofacitinib for CD treatment is not effective for inducing clinical or endoscopic remission (Dell’Avalle et al., 2022). However, JAK inhibitors increase the risk of infections, such as herpes zoster, as well as the likelihood of cardiovascular diseases, cancer, and thrombotic events (Winthrop, 2017; Olivera et al., 2020; Clarke et al., 2021). The Pharmacovigilance Risk Assessment Committee of the EMA advises that JAK inhibitors should only be used when no other appropriate treatment options are available, particularly in patients over the age of 65, those at risk of cardiovascular problems, smokers, and individuals at increased risk of cancer. Future research should focus on optimizing the selection of CD treatment medications, evaluating the efficacy and safety of combined use of biologic agents and JAK inhibitors, developing new JAK inhibitors, and investigating the safety of JAK inhibitors. These directions will guide the optimization of treatment strategies and the development of new therapeutic approaches.

This study has certain unavoidable limitations. First, all information was sourced from the WoSCC, and only English-language documents were included. With substantial contributions from Germany, France, and Italy in this field, we excluded 880 publications written in German, French, and Italian. Hence, this information might not reflect the entirety of research on CD treatment. Then, some authors not only list the school of medicine as their institution but also list the university that houses the school of medicine as a second institution. This led to the same institution being listed twice among the top 10 most productive institutions, causing a bias in the assessment of influence. Second, despite our efforts to ensure that the search criteria encompassed all pertinent literature, the risk of omissions persisted. Third, many top leaders moved between different center countries and changed their affiliations over time, which may have influenced the consistency and tracking of contributions in our analysis. Forth, the citation index is related to the date of publication. Recent literature may not have accumulated enough citations, which could affect our analysis.

## 5 Conclusion

This study demonstrated the rapid development and significant progress in the field of CD treatment over the past 2 decades. The United States has made the most significant contributions in this field and leads by a large margin in the number of published papers. The University of California System ranks first in the number of published papers, standing out among research institutions. In the first period, the focus within this field was analyzing the application and safety of anti-TNF drugs in CD. In the second period, the hotspot for CD treatment was the optimization of CD treatment plans. Currently, the main research is centered on therapies targeting alternative pathways to anti-TNF agents.

## Data Availability

The original contributions presented in the study are included in the article/Supplementary Material, further inquiries can be directed to the corresponding authors.
